# Stereotactic depth electrode placement for chronic subthreshold cortical stimulation: surgical technique video

**DOI:** 10.3171/2024.4.FOCVID2422

**Published:** 2024-07-01

**Authors:** Daniel Jeremiah Harrison, Soliman Oushy, Nicholas M. Gregg, Brian N. Lundstrom, Jamie J. Van Gompel

**Affiliations:** Departments of 1 Neurological Surgery and; 2 Neurology, Mayo Clinic, Rochester; and; 3 Mayo Clinic Alix School of Medicine, Mayo Clinic, Rochester, Minnesota

**Keywords:** epilepsy, neuromodulation, subthreshold, stimulation, cortical, chronic

## Abstract

Neurostimulation is an increasingly common treatment option for medically intractable epilepsy. SANTE (Stimulation of the Anterior Nucleus of the Thalamus for Epilepsy) and Responsive Neurostimulation (RNS) System are landmark neurostimulation trials that utilized either duty cycle or a responsive stimulation paradigm. A seizure-free outcome is rarely observed with responsive and duty cycle neurostimulation devices. Chronic subthreshold cortical stimulation (CSCS) is a promising treatment for adult drug-resistant epilepsy involving eloquent cortex and has demonstrated safety and efficacy. Herein, the authors describe the surgical technique as well as details of stimulation programming involved in CSCS placement to facilitate the adoption of this promising treatment.

The video can be found here: https://stream.cadmore.media/r10.3171/2024.4.FOCVID2422

## Transcript

### 0:24 Patient History.

Our patient is a 21-year-old right-handed woman with intractable focal epilepsy. Seizure onset occurred at 9 years of age. Primary seizure semiology is characterized by left head turn and hypermotor bicycling movements occurring 20 times monthly, often arising from sleep. Rarely seizures can progress to generalized tonic-clonic seizure activity.

### 0:46 Diagnostic Test.

MRI revealed a left superior frontal sulcation anomaly. PET was notable for subtle hypermetabolism in the left frontal and cingulate region, and MEG demonstrated a cluster of dipoles in the left frontal head region. Video-EEG monitoring recorded six left frontocentral onset seizures arising from sleep.

Further monitoring was completed with stereo-EEG targeting the hypothesized seizure onset region. Seizure onset was recorded from the LE and LN and LM contacts in the primary motor and premotor SMA region. Frequent interictal epileptic form activity was recorded from LE and LN contacts. Here is a seizure, seen arising from LE and LN contacts.

sEEG cortical stimulation mapping was notable for eloquent cortex colocalizing with the seizure onset zone. Stimulation of LE, LN, and LM contacts resulted in right hand and face motor activation.

Given colocalization of seizure onset zone and eloquent cortex, a trial of therapeutic stimulation was delivered through stereo-EEG hardware. Here, we show the device used to deliver stimulation through the sEEG contacts. From left to right, we see a clinician programmer, a clinical telemetry module, and the external neurostimulator. The ENS outputs to a custom cable terminating in 16 contacts that are connected to sEEG hardware via stackable touch-proof jumper cables.

During therapeutic stimulation there was a suppression of interictal discharges. Note a reduction in interictal discharges between the 2-Hz stimulation artifact following discontinuation of stimulation on the left side of the EEG tracing. We can see a transient suppression of interictal discharges.

### 3:06 Rationale for Procedure.

So, in this approach, a diffuse seizure onset zone involving multiple eloquent locations and successful prestimulation during sEEG predicted a good outcome with multiple therapies; however, CSCS (chronic or ongoing subthreshold cortical stimulation) was recommended to the patient. We believe this was predicted by the EEG monitoring. The benefits of decreased seizure frequency, preserved neurological function, and generally reversible if unsuccessful were utilized in this particular situation. Although all the listed therapies here are options, chronic subthreshold cortical stimulation was felt to be in the patient’s best interest through the eloquent onset area.

### 3:48 Cranial Procedure Initiation.

Here the patient is placed in a Leksell frame and coregistered with Stealth stereotaxis. We identify our entry points and align these on our subgaleal entry points. We then, in the locations where we’re targeting our cortical sites, we affix dog bones to accept our electrodes. We then align the precision aiming device. A cannula is then placed just like we do sEEG. We drill through the skull. And then a customized lead delivery device is screwed into the cannula that allows typical targeting cannula with inner diameters of 1.4 mm to be placed to increase the accuracy of the patient’s implant. Once this is done, the inner stylet is removed. We then measure from the back of this cannula. We also measure at the skull to identify the depth of the lead. The lead is then placed into the cortex. We then identify on fluoroscopy the exact location. The lead is then held at the skull, the cannula withdrawn, and it is secured with our dog bone, and then the excess wire is tied down to have galea coverage over our openings.

### 5:09 Cranial Closure.

Once all of our electrodes have been placed, we then tag these with either a series of ties in order to identify our implants when hooking this up to the battery. This is then copiously irrigated and, of course, closed in multiple layers.

### 5:27 Neurostimulation Placement.

We then go to the subclavicular region on that side, make a small incision, plan two fingerbreadths below the clavicle. We then open and make a subfascial battery pocket.

We then tunnel down from the superior cranial incision twice with two separate passers in order to allow electrodes to be passed up from the inferior incision to the superior incision. In this particular circumstance, 4 electrodes are assembled to a singular Intellis battery. The Intellis battery is a pain battery that would allow the stimulation of all 16 electrodes if possible, or multiple, or more complex stimulation parameters.

In this circumstance, the DBS intracranial leads have to be assembled to spine lead extensions, which have very tight occlusions. You have to be very careful when passing these into these areas in order not to break the electrodes. Once this is done, as typical, the booty is passed over and then secured with suture.

We then, of course, check intraoperative impedances in order to determine if the assembly is working correctly. And we again make sure that our electrode assembly is correct.

### 6:46 Wound Closure.

These are then copiously irrigated, and the wounds are closed in multiple layers in order to again prevent infection.

### 7:02 Disease Background.

Neurostimulation for medically intractable epilepsy typically consists of either a duty cycle or responsive stimulation paradigms.^1–5^ However, a seizure-free outcome is rarely observed with either of those mechanisms or approaches.^4,5^ Chronic subthreshold cortical stimulation, or what we call CSCS, aims to suppress interictal cortical epileptiform discharges by using continuous electrical stimulation. Unlike RNS, CSCS does not require the use of seizure detection algorithms, which sometimes lacks specificity. Also, CSCS provides continuous stimulation, which is advantageous over the duty cycle simulation paradigms where stimulation is often off for the majority of the time.^6^ Additionally, CSCS lends the possibility of corticothalamic implants, as demonstrated with this patient, compared to RNS. A trial stimulation is often required before implementation of a CSCS system using temporarily implanted hardware such as depth electrodes.

In summary, CSCS is a safe and effective alternative treatment for drug-resistant epilepsy, especially those involving eloquent cortex, and has demonstrated safety and efficacy.^6–9^

### 8:14 Evidence.

Although the literature is relatively sparse regarding investigations of CSCS for drug-resistant epilepsy, there are a few studies demonstrating its safety and efficacy. One study is a single-center retrospective review that compared five different neuromodulation strategies for treatment of drug-resistant epilepsy. These neuromodulation strategies included anterior thalamic DBS, centromedian thalamic DBS, RNS, CSCS, and VNS. The study included 159 patients. Overall, there was a 61% seizure reduction and 60% responder rate across all neuromodulation modalities. Notably, in an unadjusted pairwise comparison, the total median seizure reduction was most improved for CSCS when compared to the other neuromodulation modalities. Additionally, cortical stimulation, which includes both RNS and CSCS, was associated with an improved total median seizure reduction when compared to subcortical stimulation, which encompasses anterior thalamic DBS, centromedian thalamic nuclei DBS, and VNS, at 67% vs 52%, respectively.^10^

Another study investigating the safety and efficacy of CSCS is a case series of 10 adult patients with drug-resistant epilepsy due to various pathologies who underwent CSCS treatment. All patients experienced an uneventful postoperative course and experienced improvement in both seizure severity and frequency.^8^

In terms of the current state of CSCS availability, there are devices as in this particular paper that can be used to perform CSCS, such as the Medtronic or Boston Scientific devices.

### 9:42 Clinical Outcome.

The permanent electrodes are seen in yellow, and they are coregistered to the sEEG implant with seizure onset zone electrodes marked in red. For the permanent implant, 1 lead targeted the anterior nucleus of the thalamus, while 3 leads targeted the cortical seizure onset region. The patient has had 5 clinical seizures over 11 months following device placement, a greater than 90% seizure reduction.

## Disclosures

Dr. Lundstrom reported intellectual property (funds to Mayo Clinic) from Cadence Neuroscience during the conduct of the study; a patent pending for Cadence Neuroscience (17/240,746); intellectual property (funds to Mayo Clinic) from Seer Medical; site investigator for Medtronic EPAS and Neuroelectrics tDCS for Epilepsy; industry consultant for Epiminder, Medtronic, NeuroPace, and Philips Neuro (all funds to Mayo Clinic); and educational support from DIXI Medical. Dr. Van Gompel reported grants from Medtronic (investigator for the Medtronic EPAS trial, SLATE trial, and Mayo Clinic Medtronic NIH Public Private Partnership [UH3-NS95495]) and a consulting contract outside the submitted work; a patent for Cadence Neuroscience with royalties paid from named inventor for intellectual property licensed to Cadence Neuroscience, which is co-owned by Mayo Clinic; and stock ownership and a consulting contract with NeuroOne.

## Author Contributions

Primary surgeon: Van Gompel. Assistant surgeon: Oushy. Editing and drafting the video and abstract: all authors. Critically revising the work: all authors. Reviewed submitted version of the work: all authors. Approved the final version of the work on behalf of all authors: Van Gompel. Supervision: Van Gompel.

## Supplemental Information

### Patient Informed Consent

The necessary patient informed consent was obtained in this study.
